# Long term follow-up of heart rate variability in healthcare workers with mild COVID-19

**DOI:** 10.3389/fneur.2024.1403551

**Published:** 2024-05-17

**Authors:** Filippo Liviero, Maria Luisa Scapellato, Anna Volpin, Monica Battistella, Laura Fabris, Laura Brischigliaro, Franco Folino, Angelo Moretto, Paola Mason, Sofia Pavanello

**Affiliations:** ^1^Department of Cardiac, Thoracic, Vascular Sciences and Public Health, University of Padova, Padova, Italy; ^2^Occupational Medicine Unit, University Hospital of Padova, Padova, Italy

**Keywords:** SARS-CoV-2, cardiac autonomic imbalance, sympathetic heart modulation, vagal tone, autonomic nervous system, TRPV1/A1, health surveillance visit, COVID-19 symptoms

## Abstract

**Introduction:**

Prior investigations into post-COVID dysautonomia often lacked control groups or compared affected individuals solely to healthy volunteers. In addition, no data on the follow-up of patients with SARS-CoV-2-related autonomic imbalance are available.

**Methods:**

In this study, we conducted a comprehensive clinical and functional follow-up on healthcare workers (HCWs) with former mild COVID-19 (group 1, n = 67), to delineate the trajectory of post-acute autonomic imbalance, we previously detected in a case–control study. Additionally, we assessed HCWs for which a test before SARS-CoV-2 infection was available (group 2, n = 29), who later contracted SARS-CoV-2, aiming to validate findings from our prior case–control investigation. We evaluated autonomic nervous system heart modulation by means of time and frequency domain heart rate variability analysis (HRV) in HCWs during health surveillance visits. Short-term electrocardiogram (ECG) recordings, were obtained at about 6, 13 months and both at 6 and 13 months from the negative SARS-CoV-2 naso-pharyngeal swab (NPS) for group 1 and at about 1-month from the negative NPS for group 2. HCWs who used drugs, had comorbidities that affected HRV, or were hospitalized with severe COVID-19 were excluded.

**Results:**

Group 1 was split into three subgroups clinically and functionally followed at, about 6 months (subgroup-A, *n* = 17), 13 months (subgroup-B, *n* = 37) and both at 6 and 13 months (subgroup-C, *n* = 13) from the negative SARS-CoV-2 NPS. In subgroup-A, at 6-month follow-up compared with baseline, the spectral components in the frequency domain HRV parameters, showed an increase in normalized high frequency power (nHF) (*t* = 2.99, *p* = 0.009), a decrease in the normalized low frequency power (nLF) (*t* = 2.98, *p* = 0.009) and in the LF/HF ratio (*t* = 3.13, p = 0.006). In subgroup B, the comparison of the spectral components in the frequency domain HRV parameters, at 13-month follow-up compared with baseline, showed an increase in nHF (*t* = 2.54, *p* = 0.02); a decrease in nLF (*t* = 2.62, *p* = 0.01) and in the LF/HF ratio (*t* = 4.00, *p* = 0.0003). In subgroup-C, at both 6 and 13-month follow-ups, the spectral components in the frequency domain HRV parameters were higher than baseline in nHF (*t* = 2.64, p = 0.02 and (*t* = 2.13, *p* = 0.05, respectively); lower in nLF (*t* = 2.64, p = 0.02 and (*t* = 2.13, p = 0.05, respectively), and in LF/HF (*t* = 1.92, p = 0.08 and (*t* = 2.43, *p* = 0.03, respectively). A significant proportion of HCWs reported persistent COVID-19 symptoms at both the 6 and 13-month follow-ups, seemingly unrelated to cardiac autonomic balance. In group 2 HCWs, at 1-month follow-up compared with baseline, the spectral components in the frequency domain HRV parameters, showed a decrease in nHF (*t* = 2.19, *p* = 0.04); an increase in nLF (*t* = 2.15, *p* = 0.04) and in LF/HF (*t* = 3.49, *p* = 0.002).

**Conclusion:**

These results are consistent with epidemiological data suggesting a higher risk of acute cardiovascular complications during the first 30 days after COVID-19. The SARS-CoV-2 associated autonomic imbalance in the post-acute phase after recovery of mild COVID-19 resolved 6 months after the first negative SARS-CoV-2 NPS. However, a significant proportion of HCWs reported long-term COVID-19 symptoms, which dot not seems to be related to cardiac autonomic balance. Future research should certainly further test whether autonomic imbalance has a role in the mechanisms of long-COVID syndrome.

## 1 Introduction

The global impact of Coronavirus disease 2019 (COVID-19), stemming from severe acute respiratory syndrome coronavirus 2 (SARS-CoV-2), has been staggering, with nearly 7 million deaths attributed to the virus worldwide (1). Several studies have reported an increased risk of short-term (2–5) and long-term (4, 6–8) cardiovascular disease and mortality after SARS-CoV-2 infection. In response to the urgent need for understanding post-acute effects of COVID-19, our recent investigation delved into the clinical and functional follow-up of previously examined individuals, aimed at elucidating the trajectory of post-acute autonomic imbalance. Through symptom collection and repeated assessment of Heart Rate Variability (HRV), we ascertained the persistence of autonomic dysregulation following recovery from mild COVID-19. In addition, we aimed to determine whether Healthcare Workers (HCWs), who underwent pre-infection SARS-CoV-2 testing and later contracted the virus exhibited cardiac autonomic imbalance, thus corroborating the findings of our previous case–control study. In that study we observed an association between SARS-CoV-2 infection and post-acute autonomic imbalance, characterized by sustained sympathetic heart modulation and diminished vagal heart modulation, as reflected by reduced HRV (9). Notably, prior investigations into post-COVID dysautonomia often lacked control groups or compared affected individuals solely to healthy volunteers (10). By including fully recovered post-COVID cohorts, our study aimed to identify whether there remain autonomic residual effects of the infection. The absence of data on the follow-up of patients with SARS-CoV-2-related autonomic imbalance underscores the significance of our findings. Insights gleaned from our research may shed light on the epidemiological observations of increased acute cardiovascular complications within 30 days post-SARS-CoV-2 infection (2–5), while elucidating underlying pathogenetic mechanisms of both acute COVID-19 and long-COVID. Although post-COVID dysautonomia may clinically improve over time for most patients, persistent autonomic dysfunction in select individuals necessitates ongoing clinical and functional monitoring. Utilizing assessment of HRV as a reliable and non-invasive metric for quantifying sympathetic and parasympathetic heart modulation (11), our study underscores the importance of characterizing cardiac autonomic function, particularly in the post-recovery phase of COVID-19. In this study, we conducted a comprehensive clinical and functional follow-up on HCWs, previously categorized as cases, to delineate the trajectory of post-acute autonomic imbalance. Additionally, we assessed HCWs previously considered as controls who later contracted SARS-CoV-2, aiming to validate findings from our prior case–control investigation.

## 2 Materials and methods

### 2.1 Study design and population

**Figure 1:**
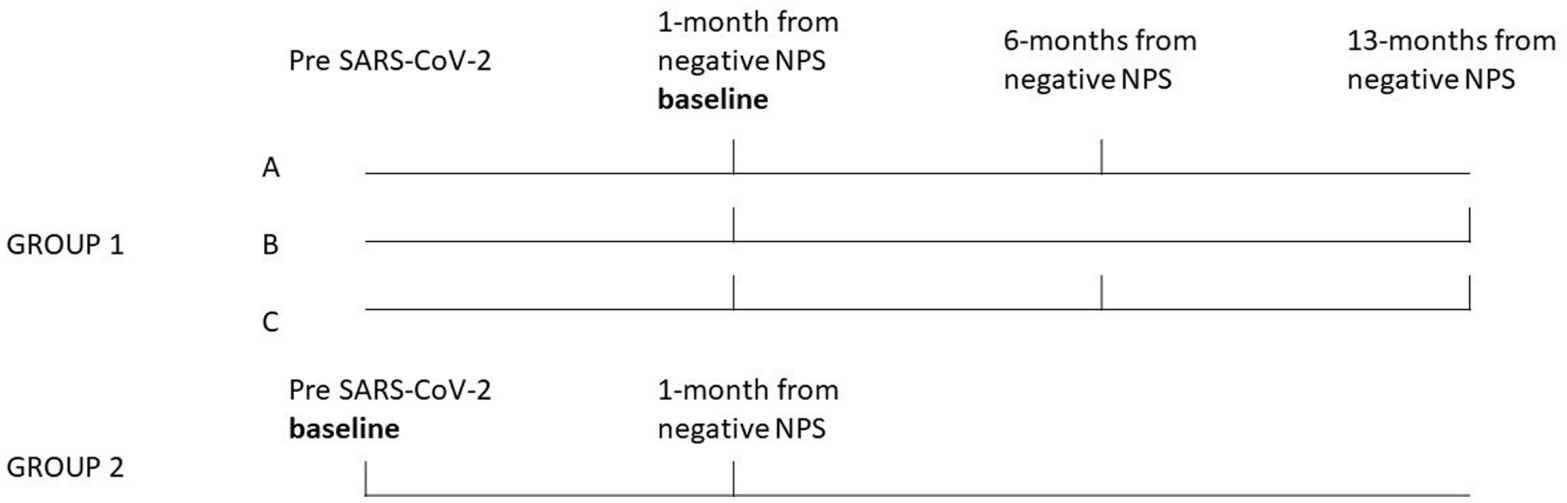
Study design. The bold vertical lines indicate the period during which clinical follow-up and HRV tests were conducted for each group and subgroup of HCWs. Group 1 was split into three subgroups clinically and functionally followed at, about, 6 months (subgroup-A, *n* = 17), 13 months (subgroup-B, *n* = 37) and both at 6 and 13 months (subgroup-C, *n* = 13) from the negative SARS-CoV-2 NPS. The results of HRV follow-up measurements for these subgroups, were compared to the baseline. Group 2 (*n* = 29), consisted of HCWs for which a test before SARS-CoV-2 infection was also available (the baseline for this group), the results of the HRV follow-up measurements conducted at about 1-month from the negative NPS were compared to the baseline.

HCWs employed at the University Hospital of Padova, who had previously participated in another study (9), were summoned to partake in a clinical and functional follow-up. This follow-up, involving the repetition of HRV assessments, was integrated into routine health surveillance procedures mandated by legislative decree 81/08 and European Community Directive 90/679. The study design is reported in Figure 1.

Group 1 (*n* = 67) consisted of HCWs who had a SARS-CoV-2 infection (between October 2020 and September 2022) and were previously studied (9) with HRV tests conducted in the post-acute phase, i.e., about 30 days from the negative SARS-CoV-2 naso-pharyngeal swab (NPS), the baseline. Group 1 was split into three subgroups clinically and functionally followed at, about, 6 months (subgroup-A, *n* = 17), 13 months (subgroup-B, *n* = 37) and both at 6 and 13 months (subgroup-C, *n* = 13) from the negative SARS-CoV-2 NPS. The results of HRV follow-up measurements of subgroups A, B and C were compared to the baseline. Group 2 (*n* = 29), consisted of HCWs for which a test before SARS-CoV-2 infection was also available (the baseline for this group), since they have been considered as controls in our previous study (9), but contracted SARS-CoV-2 subsequently, between August 2021 and December 2022. Also for this group the results of 1-month HRV follow-up measurements from the negative NPS, were compared to the baseline. Subjects were excluded if they had active COVID-19 infection, and history of severe SARS-CoV-2 infection (i.e., need to hospitalization or home oxygen treatment, and severe respiratory or other major organ involvements) and if they were affected or have a history of diseases interfering with the analysis. Moreover, subjects using drugs interfering with the HRV measurement (i.e., beta-blockers, calcium channel blockers, inhaled or oral beta-mimetics, theophylline, or other drugs with potential chronotropic effects), were excluded. HCWs who regularly work a night shift (i.e., from 8 p.m. to 6 a.m. in our Hospital) at least 5 times a month have been defined as night workers. Symptoms were collected using the COVID-19 rapid guideline (12), at the follow-up visits (i.e., about 1, 6 and 13 months after the negative SARS-CoV-2NPS). The study was approved by the local Research Ethics Committee (Protocol number = 267n/AO/22) and conducted in accordance with the ethical principles stated in the “Declaration of Helsinki.”

### 2.2 Assessment of autonomic heart modulation, HRV analysis and blood pressure

HRV was assessed as previously described (9); briefly, all study subjects were instructed to avoid from smoking, and to stop coffee and alcohol intake for 2 h and 48 h, respectively. They should have had sufficient (at least 8 h) rest, as well as not having worked the night shift on the night before the test was performed. HRV was assessed by short-term electrocardiogram (ECG), performed in a supine position, under physiologically stable conditions, and using a device connected to the patient via two electrodes. For group 1, ECG was recorded during follow-up visits (i.e., at 6 and 13 months after the negative SARS-CoV-2NPS). For group 2, ECG was recorded after a negative NPS for SARS-CoV-2 and after symptoms disappeared (since at least three days). HRV data were acquired by a Bluetooth acquisition system (BT16 Plus, FM, Monza, Italy). ECG was recorded between 9 and 14 a.m. at rest under ideal temperature conditions, for at least 5 min. HRV was analyzed using Kubios HRV software (ver. 3.3) (13). Normal and aberrant complexes were identified and all of the adjacent intervals between normal beats over 5 min intervals were considered. As previously described (9), we analyzed the spectral components (HRV frequency domain variables) as the absolute values of power (ms^2^) using an autoregressive modeling based method (AR spectrum), applying the default value of 16 for the model order (11). The main spectral components considered were very low frequency (VLF), low frequency (LF), high-frequency (HF) and the LF/HF ratio. The area under the curve of the spectral peaks within the frequencies 0.01–0.4, 0.01–0.04, 0.04–0.15, and 0.15–0.40 Hz was defined as the total power (TP), very low-frequency power (VLF), low-frequency power (LF), and high-frequency power (HF), respectively. LF and HF, were normalized to the total power within the frequency range of 0.01–0.4 Hz. The normalized low-frequency power (nLF = LF/TP) represents an index of combined sympathetic and vagal modulation (14) as well as a baroreflex index (15, 16), while the normalized HF power (nHF = HF/TP) corresponds to an index of vagal heart modulation. The low/high-frequency power ratio (LF/HF) is thus an index of sympathovagal balance. Time domain measures included the standard deviation of normal-to-normal RR intervals (SDNN), the root mean square of successive RR interval differences (RMSSD). SDNN is considered as an estimate of the overall HRV which corresponds to the total power in the frequency domain. RMSSD is considered as an estimate of short-term components of HRV and correlates with HF in the frequency domain (11). Office blood pressure was measured once using an Omron 705IT electronic device (Omron Healthcare Europe, the Netherlands), while the patient has been lying calmly for at least 5 min, in line with the 2023 European Society of Hypertension (17).

### 2.3 Statistical analysis

Statistical analyses were performed with the use of Minitab, LLC, version 18.0. The Kolmogorov-Smirnov test was performed to evaluate whether the variables were normally distributed. Continuous variables were presented as means ± SE or median (IQR 25–75) and categorical variables as frequency. Data with a wide dispersion were expressed in log transformed values. For continuous data, Student’s paired *t*-test, two-sample *t*-test and One-way ANOVA test were used when indicated. Fisher exact test was used to determine whether a statistically significant association exists between two categorical variables. All *p* values less than 0.05 were considered significant. Lastly, the influence of independent variables, including age, sex, night work, body mass index, cardiac symptoms (i.e., palpitations and tachycardia), systolic and diastolic blood pressure differences (post-pre SARS-CoV-2 infection) and manual handling of loads and manual handling of patients on delta LF/HF (difference post-pre SARS-CoV-2 infection), as dependent variable, was appraised by multiple linear regression analysis.

## 3 Results

Table 1 shows characteristics of the study subjects.

**Table 1:** Characteristics of the study population.

Study variables	Group 1 (*n* = 67)				Group 2 (*n* = 29)
	Subgroup-A (*n* = 17)	Subgroup-B (*n* = 37)	Subgroup-C (*n* = 13)	*p*-value	
Follow up period, days	188;(161–225)		201;(161–249)	0.74	26;(17–34.5)
		383;(349–504)	376;(348–394)	0.08	
Age, years	49.8 ± 8.41	48.5 ± 10.2	51.7 ± 6.64	0.55	45.9 ± 10.1
Male gender, *n* (%)	3 (17.6%)	10 (27%)	3 (23%)	0.75	7 (24.1%)
Body mass index, kg/m^2^	23.9 ± 4.22	25.3 ± 4.93	23.9 ± 4.53	0.49	23.3 ± 3.84
Night shift workers, *n* (%)	3 (17.7%)	14 (37.8%)	2 (15.4%)	0.16	9 (31.0%)
Vaccinated HCWs at follow-up visit, *n* (%)	16 (94.1%)	36 (97.3%)	13 (100%)	0.53	28 (96.6%)
Acute phase disease duration, days	11; (9–16)	14;(9.5–20.5)	11;(9–15.5)	0.25	10;(8–11)

Values are given as *n* and %, mean (± standard error) or median (IQR 25–75). Statistical comparisons were made by two-sample *t*-test and One-way ANOVA test to check statistical differences between two groups and three groups, respectively. Level of significance < 0.05.

Regarding group 1 subgroups A, B and C, the median elapsed time from the negative SARS-CoV-2 NPS to ECG recording was: 188 days (IQR 161–225), 383 days (IQR 349–504) and 201 days (IQR 161–249) and 376 days (IQR 348–394), respectively. The characteristics of the study population did not significantly differ between the three subgroups (Table 1). For group 2, the median elapsed time from the negative SARS-CoV-2 NPS to ECG recording was 26 days (IQR 17–34.5).

### 3.1 Results of follow-up among group 1 HCWs

**Figure 2:**
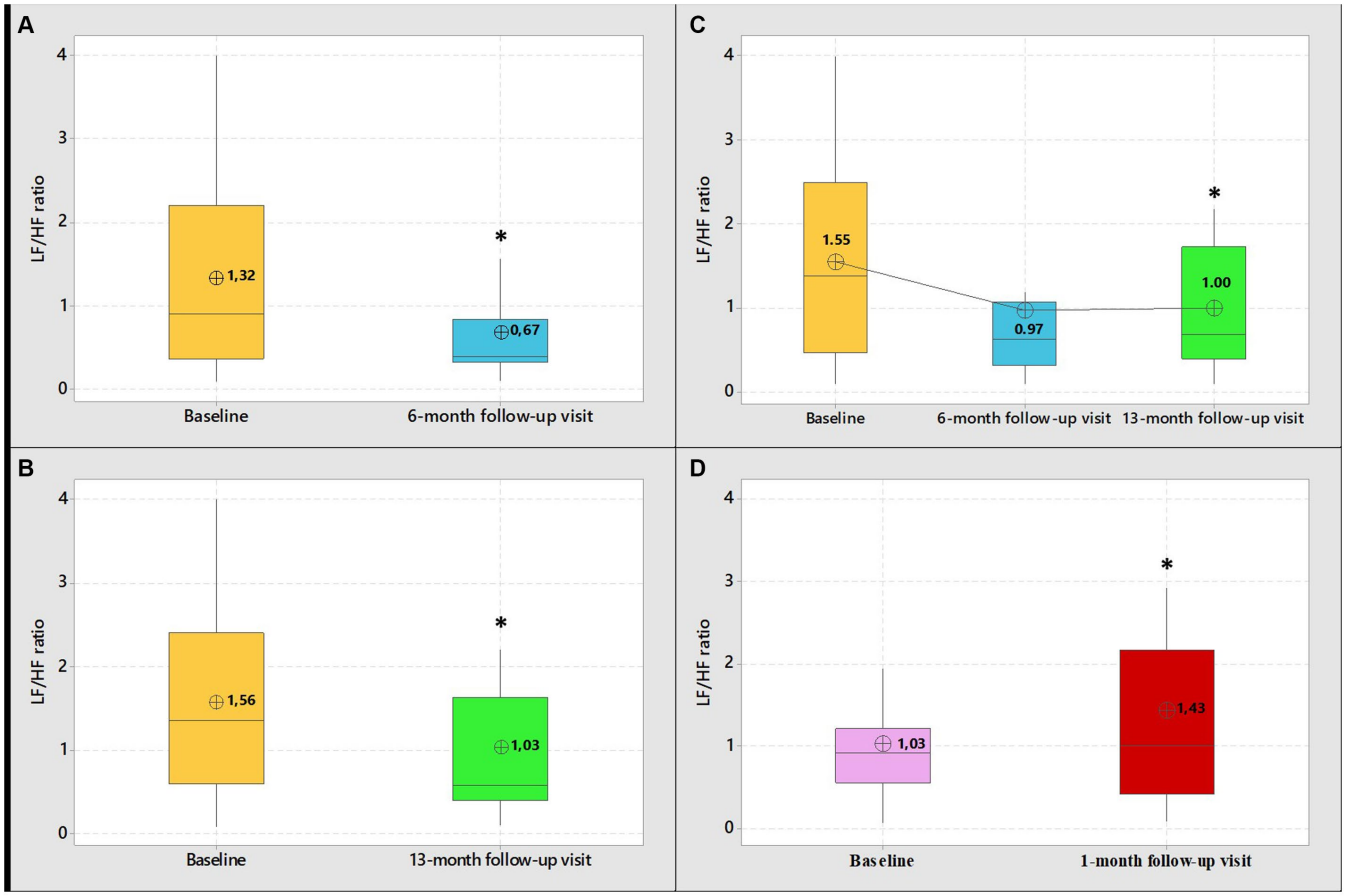
Boxplot graphical representation of LF/HF ratio among group 1 HCWs (subgroups-A-B-C) at baseline and at 6-month follow-up visit **(A)** 13-month follow-up visit **(B)** and 6 and 13-month follow-up visit **(C)**. Boxplot graphical representation of LF/HF ratio among group 2 HCWs at baseline and at 1-month follow-up visits **(D)**. In box plots, the boundary of the box closest to zero indicates the 25th percentile, the line within the box marks the median, and the boundary of the box farthest from zero indicates the 75th percentile. The whiskers (error bars) above and below the box indicate the 95th and 5th percentiles. The circle with the inner cross indicates the mean value. *Student’s paired *t*-test, level of significance <0.05.

Table 2, shows the frequency and time domain analysis of HRV and systolic and diastolic blood pressure in group 1 HCWs subgroup-A (*n* = 17), in both visits (baseline and 6-month follow-up). At 6-month follow-up compared with baseline, the spectral components in the frequency domain HRV parameters, showed an increase in normalized high frequency power (nHF) (*t* = 2.99, *p* = 0.009), a decrease in the normalized low frequency power (nLF) (*t* = 2.98, *p* = 0.009) and in the LF/HF ratio (*t* = 3.13, *p* = 0.006), (Figure 2A). Among time domain parameters, no statistically significative differences were registered for SDNN and RMSSD. Diastolic blood pressure resulted significantly lower at 6-month follow-up compared with baseline (*t* = 2.68, *p* = 0.02). Systolic blood pressure and mean HR that were in the range of normal resting values in both visits did not change at 6-month follow-up compared with baseline (Table 2).

**Table 2:** Frequency and time domain analysis of HRV and systolic and diastolic blood pressure values (mean ± standard error), in group 1 HCWs subgroup-A, at baseline and at 6-month follow-up visit.

Variable	Baseline	6-month follow-up	*p*-value
nLF	47.1 ± 22.9	34.8 ± 16.4	0.009 **
nHF	52.8 ± 22.8	65.2 ± 16.3	0.009 **
LF/HF	1.32 ± 1.13	0.67 ± 0.62	0.006 **
SDNN^a^	1.38 ± 0.20	1.39 ± 0.20	0.97
RMSSD^a^	1.35 ± 0.25	1.41 ± 0.23	0.46
Mean HR, bpm	73.2 ± 10.2	68.7 ± 10.5	0.10
Systolic blood pressure, mmHg	128.8 ± 14.2	125.3 ± 16.4	0.49
Diastolic blood pressure, mmHg	83.2 ± 5.85	80.0 ± 5.86	0.02 *

nLF, normalized low frequency; nHF, normalized high frequency; LF/HF, low/high-frequency ratio; SDNN, standard deviation of normal-to-normal R-R intervals; RMSSD, root mean square of successive RR interval differences. Student’s paired *t*-test, level of significance *p < 0.05 and **p < 0.01.

a Log transformed values. Bold values indicate statistically significant results.

Table 3, shows frequency and time domain analysis of HRV and systolic and diastolic blood pressure in group 1 HCWs subgroup-B (*n* = 37), in both visits. The comparison of the spectral components in the frequency domain HRV parameters, at 13-month follow-up compared with baseline, showed an increase in nHF (*t* = 2.54, *p* = 0.02); a decrease in nLF (*t* = 2.62, *p* = 0.01) and in the LF/HF ratio (*t* = 4.00, *p* = 0.0003) (Figure 2B). Among time domain parameters, no statistically significative differences were registered for SDNN, between the two visits. Regarding RMSSD, the mean value at 13-month follow-up was higher than baseline (*t* = 2.30, *p* = 0.03). In addition, systolic and diastolic blood pressure values did not change. Mean HR at 13-month follow-up was lower than baseline (*t* = 3.24, *p* = 0.003). However, blood pressure and mean HR were in the range of normal resting values in both visits (Table 3).

**Table 3:** Frequency and time domain analysis of HRV and systolic and diastolic blood pressure values (mean ± standard error), in group 1 HCWs subgroup-B, at baseline and at 13-month follow-up visit.

Variable	Baseline	13-month follow-up	*p*-value
nLF	52.7 ± 19.6	44.9 ± 20.3	0.01 *
nHF	47.3 ± 19.6	55.0 ± 20.2	0.02 *
LF/HF	1.56 ± 1.28	1.03 ± 0.96	0.0003 **
SDNN^a^	1.37 ± 0.22	1.40 ± 0.18	0.26
RMSSD^a^	1.33 ± 0.29	1.42 ± 0.22	0.03 *
Mean HR, bpm	73.4 ± 9.08	69.2 ± 8.85	0.003 *
Systolic blood pressure, mmHg	130.0 ± 15.5	126.4 ± 15.8	0.18
Diastolic blood pressure, mmHg	82.3 ± 7.42	80.4 ± 9.53	0.24

nLF, normalized low frequency; nHF, normalized high frequency; LF/HF, low/high-frequency ratio; SDNN, standard deviation of normal-to-normal R-R intervals; RMSSD, root mean square of successive RR interval differences. Student’s paired *t*-test, level of significance *p < 0.05 and **p < 0.01.

a Log transformed values. Bold values indicate statistically significant results.

Table 4, show frequency and time domain analysis of HRV and systolic and diastolic blood pressure values, in group 1 HCWs subgroup-C (*n* = 13), at baseline, 6 and 13-month follow-up visits. At both 6 and 13-month follow-ups the spectral components in the frequency domain HRV parameters were higher than baseline in nHF (*t* = 2.64, *p* = 0.02 and *t* = 2.13, *p* = 0.05, respectively); lower in nLF (*t* = 2.64, *p* = 0.02 and *t* = 2.13, *p* = 0.05, respectively), and in LF/HF (*t* = 1.92, *p* = 0.08 and *t* = 2.43, *p* = 0.03, respectively) (Figure 2C). Among time domain parameters, no differences were registered for SDNN and RMSSD, between the two visits. In addition, systolic and diastolic blood pressure values did not significantly change at both 6 and 13-month follow-ups compared with baseline. Mean HR at 13-month follow-up was lower than baseline (*t* = 2.30, *p* = 0.04). However, blood pressure and mean HR were in the range of normal resting values in both visits (Table 4).

**Table 4:** Frequency and time domain analysis of HRV and systolic and diastolic blood pressure values (mean ± standard error), in group 1 HCWs subgroup-C, at baseline and at 6 and 13-month follow-up visits.

Variable	Baseline	6-month follow-up	*p*-value	13-month follow-up	*p*-value
nLF	51.9 ± 22.2	39.2 ± 20.6	0.02*	44 ± 19.1	0.05
nHF	47.9 ± 22.1	60.8 ± 20.5	0.02*	56 ± 19.1	0.05
LF/HF	1.55 ± 1.18	0.97 ± 1.14	0.08	1 ± 0.70	0.03*
SDNN^a^	1.36 ± 0.18	1.34 ± 0.15	0.82	1.35 ± 0.22	0.83
RMSSD^a^	1.32 ± 0.25	1.35 ± 0.17	0.65	1.36 ± 0.24	0.48
Mean HR, bpm	73.5 ± 11.49	70.5 ± 10.36	0.32	67.6 ± 9.02	0.04*
Systolic blood pressure, mmHg	129.2 ± 14.6	128.1 ± 17.9	0.85	122.3 ± 13.5	0.19
Diastolic blood pressure, mmHg	83.5 ± 6.25	80.4 ± 6.60	0.05	80.4 ± 5.58	0.15

nLF, normalized low frequency; nHF, normalized high frequency; LF/HF, low/high-frequency ratio; SDNN, standard deviation of normal-to-normal R-R intervals; RMSSD, root mean square of successive RR interval differences. *Student’s paired *t*-test, level of significance *p* < 0.05.

a Log transformed values. Bold values indicate statistically significant results.

### 3.2 Symptoms

**Figure 3:**
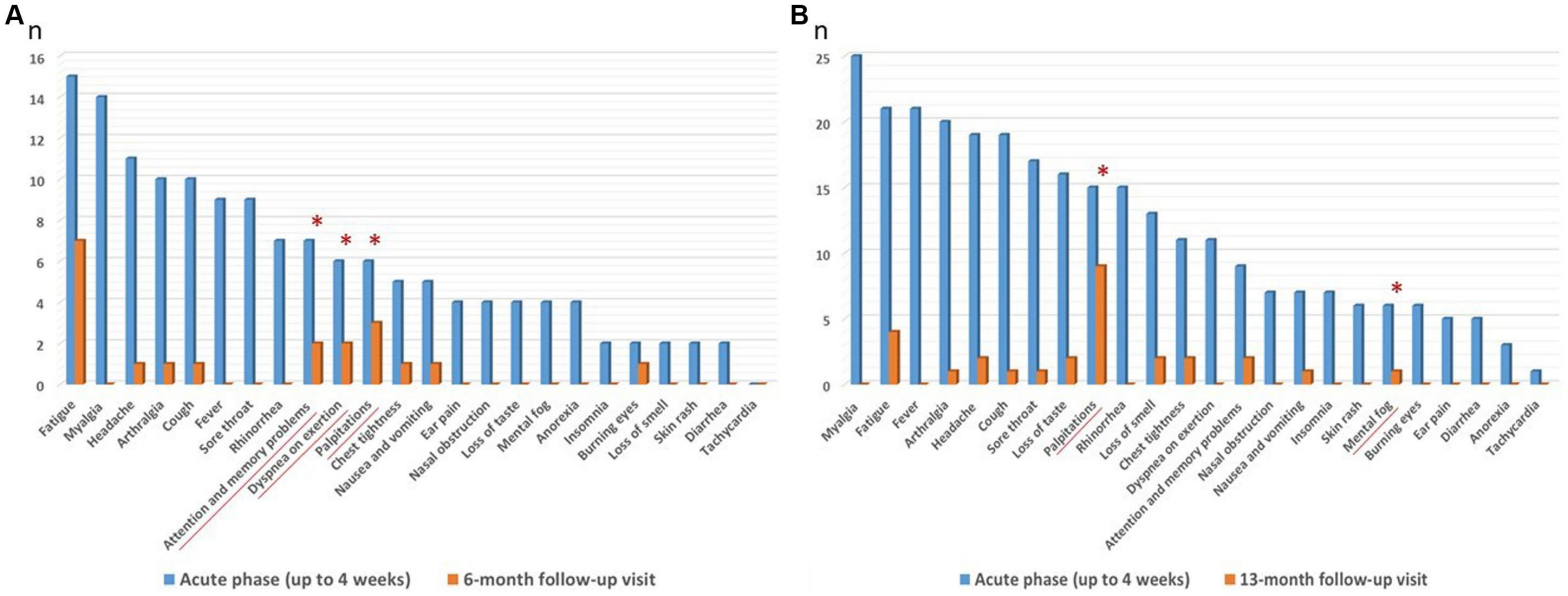
**(A,B)** Most commonly reported symptoms (blue columns) by subgroup-A and B HCWs, during COVID-19 acute phase (i.e., up to 4 weeks after the start of confirmed COVID-19) and persisting (orange columns) at 6 (subgroup-A, *n* = 17) and 13 (subgroup-B, *n* = 37) month follow-up visits. *Most significative persistent symptoms *p* > 0.05, at follow-up visits. Values are given as number of subjects (*n*).

At the follow-up visits, all subjects reported mild SARS-CoV-2 symptoms. For subgroup-A HCWs, acute phase (i.e., up to 4 weeks after the start of confirmed COVID-19) most commonly reported symptoms were: fatigue (88.2%), myalgia (82.3%), headache (64.7%), arthralgia (58.9%), cough (58.9%), fever (52.9%) and sore throat (52.9%). Overall, in this subgroup at 6-month follow-up visit, 10 HCWs (58.8%) were asymptomatic, 7 HCWs (41.2%) continued to complain at least 1 symptom (whose 1 HCW with one symptom, 1 HCW with two symptoms and 5 HCWs with three or more symptoms). At 6-month follow-up visit the most persistent symptoms (*p* > 0.05), were palpitations (17.6%), dyspnea on exertion (11.8%) and attention and memory problems (11.8%) (Figure 3A, Supplementary Table S1). For subgroup-B HCWs, acute phase (i.e., up to 4 weeks after the start of confirmed COVID-19) most commonly reported symptoms were: myalgia (67.5%), fatigue (56.8%), fever (56.8%), arthralgia (54.0%), headache (51.4%), cough (51.4%) and sore throat (45.9%). Overall, in this subgroup at 13-month follow-up visit, 23 HCWs (62.2%) were asymptomatic, 14 HCWs (37.8%) continued to complain at least 1 symptom (whose 6 HCWs with one symptom, 3 HCWs with two symptoms and 5 HCWs with three or more symptoms). The most significative persistent symptoms (*p* > 0.05), were palpitations (24.3%) and mental fog (0.03%), (Figure 3B, Supplementary Table S2).

No significative differences in the autonomic control of the heart, indexed by LF/HF among group 1 HCWs (subgroups A and B), at 6-month and 13-month functional follow-up respectively, were found between HCWs with most significative persistent symptoms vs. HCWs without significative persistent symptoms (Supplementary Table S3). Supplementary Table S4, show sex differences in the autonomic control of the heart, indexed by LF/HF among group 1 HCWs followed at baseline, 6 months (subgroup-A), 13 months (subgroup-B), and 6 and 13 months (subgroup-C), after the negative SARS-CoV-2 NPS. A significative difference between sex was reached only in subgroup-A HCWs (*n* = 17), with an increased LF/HF in males compared to females at baseline test (performed about 1 month after the negative SARS-CoV-2 NPS) (*p* = 0.02).

### 3.3 Results of follow-up among group 2 HCWs

Table 5, show frequency and time domain analysis of HRV and systolic and diastolic blood pressure values, in group 2 HCWs (*n* = 29), in both visits. At 1-month follow-up compared with baseline, the spectral components in the frequency domain HRV parameters, showed a decrease in nHF (*t* = 2.19, *p* = 0.04); an increase in nLF (*t* = 2.15, *p* = 0.04) and in LF/HF (*t* = 3.49, *p* = 0.002) (Figure 2D). Among time domain parameters, no statistically significative differences were registered for SDNN and RMSSD, between the two visits. In addition, mean HR and systolic and diastolic blood pressures did not significantly change at 1-month follow-up compared to baseline and were in the range of normal resting values in both visits (Table 5).

**Table 5:** Frequency and time domain analysis of HRV and systolic and diastolic blood pressure values (mean ± standard error), in group 2 HCWs at baseline and at 1-month follow-up visit.

Variable	Baseline	1-month follow-up	*p*-value
nLF	45.3 ± 16.6	50.4 ± 20.5	0.04 *
nHF	54.7 ± 16.6	49.5 ± 20.4	0.04 *
LF/HF	1.03 ± 0.78	1.43 ± 1.24	0.002 **
SDNN^a^	1.38 ± 0.18	1.36 ± 0.21	0.35
RMSSD^a^	1.37 ± 0.24	1.33 ± 0.29	0.37
Mean HR, bpm	72.2 ± 10.04	72.6 ± 10.80	0.82
Systolic blood pressure, mmHg	124.3 ± 10.9	121.4 ± 7.78	0.21
Diastolic blood pressure, mmHg	80.2 ± 5.43	82.1 ± 4.73	0.13

nLF, normalized low frequency; nHF, normalized high frequency; LF/HF, low/high-frequency ratio; SDNN, standard deviation of normal-to-normal R-R intervals; RMSSD, root mean square of successive RR interval differences. Student’s paired *t*-test, level of significance *p < 0.05 and **p < 0.01.

a Log transformed values. Bold values indicate statistically significant results.

**Figure 4:**
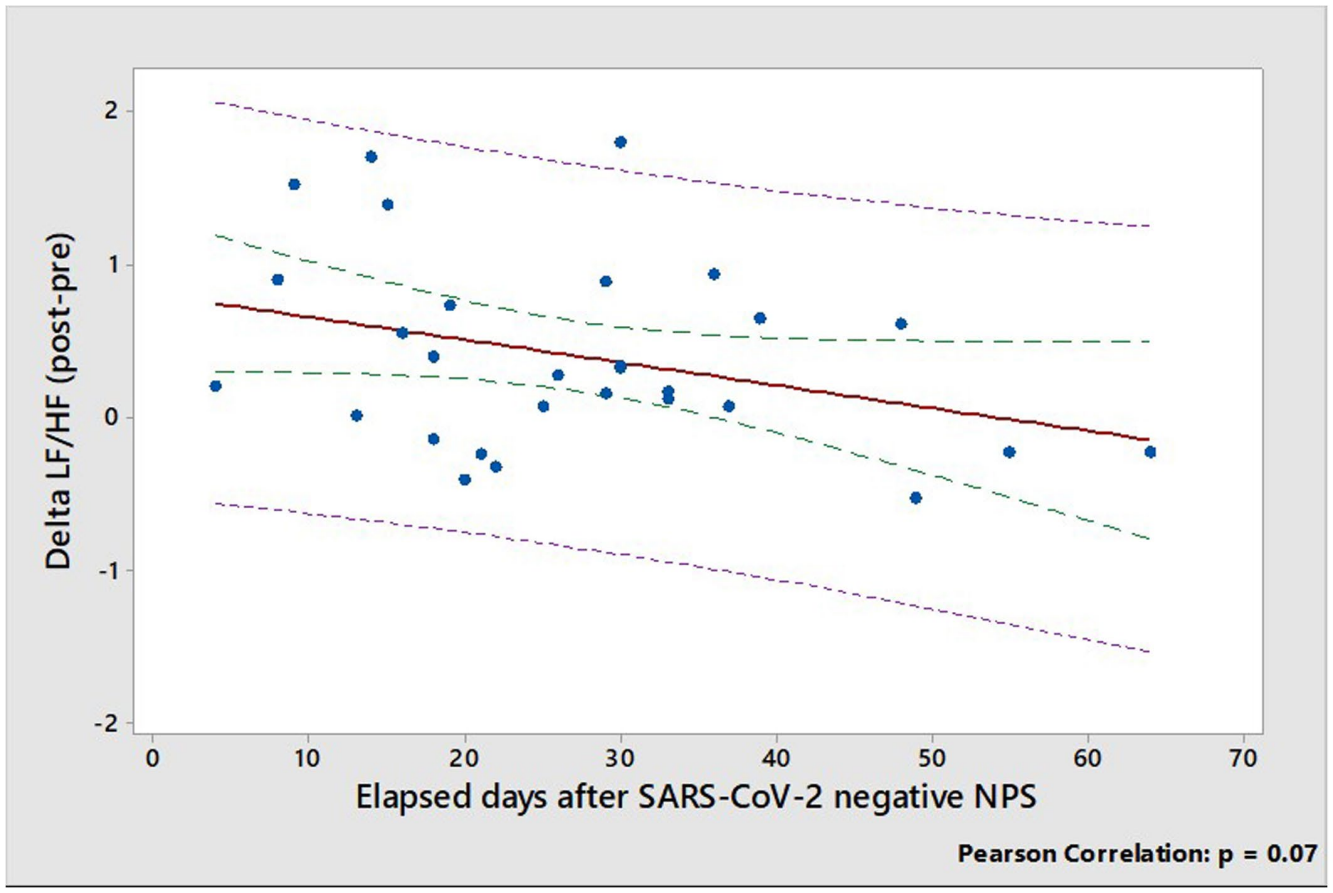
Relationship between delta LF/HF (expressed as the difference of LF/HF ratios post-pre SARS-CoV-2 infection) and elapsed days from the negative SARS-CoV-2 NPS. Dotted green and pink lines show confidence and prediction intervals, respectively.

Multiple linear regression analysis showed that the principal determinant of delta LF/HF (expressed as the difference of LF/HF ratios post-pre SARS-CoV-2 infection), is the elapsed days from the negative SARS-CoV-2 NPS (Supplementary Table S5). Indeed, delta LF/HF tends to decrease almost significantly (*r* = 0.34, *p* = 0.07), with time from the negative SARS-CoV-2 NPS, and tends to zero at about two months after SARS-CoV-2 negative NPS (Figure 4).

Supplementary Table S6, show sex differences in the autonomic control of the heart, indexed by LF/HF among group 2 HCWs at baseline (i.e., pre SARS-CoV-2 infection) and at about 1 month functional follow-up after the negative SARS-CoV-2 NPS. Interestingly, also in this group a significative difference between sex was reached only at 1-month functional follow-up with an increased LF/HF in males compared to females (*p* = 0.03).

## 4 Discussion

Our investigation into the clinical and functional follow-up of HCWs with previous mild SARS-CoV-2 infection (group 1) revealed relevant insights:

1. The autonomic cardiac regulation imbalance, characterized by increased sympathetic heart modulation and decreased vagal heart modulation, consistently resolved in all subgroups (i.e., A and C) six months after the negative SARS-CoV-2 NPS. This recovery was confirmed at the 13-month follow-up in all subgroups (i.e., B and C).
2. Mean HR remained within the normal resting range, exhibiting a decreasing trend at the 13-month follow-up compared to the post-acute phase (subgroups B and C).
3. No significant changes in systolic and diastolic blood pressure values were evidenced.
4. A significant proportion of HCWs reported persistent COVID-19 symptoms at both the 6 and 13-month follow-ups, seemingly unrelated to cardiac autonomic balance.

Furthermore, the functional follow-up of HCWs in group 2 confirmed the autonomic cardiac regulation imbalance during the post-acute phase of infection. This was characterized by increased sympathetic heart modulation (reflected by an increase in nLF and LF/HF) and decreased vagal heart modulation (evidenced by a reduction in nHF). Remarkably, the autonomic cardiac imbalance tended to resolve as early as two months after a negative SARS-CoV-2 NPS. Unlike our previous findings, no significant changes in time domain parameters (i.e., the SDNN and RMSSD methods) were registered. Regardless of the use of time-domain methods to analyze recordings of short durations, it is crucial to emphasize that frequency methods should be the preferred choice when investigating short-term recordings (11). Overall, the findings of this work seem to be more reliable because reinforced from HRV tests repeated in the same subjects, before and after infection.

Dysfunction of Autonomic Nervous System (ANS) can manifest following SARS-CoV-2 infection, and HRV emerges as a reliable and non-invasive tool to assess its integrity. Existing data, primarily from observational case–control studies, shed light on the direct impact of SARS-CoV-2 infection on HRV. A systematic review of 17 observational studies revealed a consistent and significant drop in vagal heart modulation, associated with SARS-CoV-2 infection (18). Another review, comprising 11 case–control studies on individuals recovering from acute COVID-19, indicated decreased parasympathetic heart modulation in post-COVID-19 or long-COVID individuals compared to controls (19). Notably, long-COVID individuals exhibited significantly lower HF and a higher LF/HF ratio, suggesting a potential association with reduced parasympathetic heart modulation and increased sympathetic heart modulation (20–22). Furthermore, a systematic review analyzing 22 articles focused on hospitalized patients during the acute phase of SARS-CoV-2 infection concluded that autonomic dysfunction occurs early in the disease progression (23). Although most studies affirmed HRV alterations during the acute phase, the specific involvement of sympathetic or parasympathetic pathways varied. The heterogeneity in study populations, recording tools, HRV parameters analyzed, and methodological quality underscore the need for more rigorous and accurate measurements to confirm these findings.

In our study, HRV tests were performed in the same subjects before infection and in the post-acute phase of SARS-CoV-2. This provides a more convincing evidence for the autonomic cardiac regulation imbalance, characterized by increased sympathetic heart modulation and decreased vagal heart modulation, that was previously observed in case–control design studies. The autonomic cardiac imbalance in mild cases resolved after six months, with recovery apparent as early as two months after a negative SARS-CoV-2 NPS, in certain HCWs. However, a significant percentage of HCWs reported long-term COVID-19 symptoms persisting independent from autonomic balance recovery. These individuals are now enrolled in a dedicated long-COVID study project in our center, exploring potential associations with markers of inflammation and cellular senescence, factors that may negatively impact HRV (24). In essence, our findings underscore the dynamic nature of autonomic dysregulation post-SARS-CoV-2 infection and highlight the importance of continued investigation to understand its persistence and potential clinical implications.

The substantial reduction in vagal heart modulation, as indicated by a decrease in nHF, observed in association with SARS-CoV-2 infection, resonates across various case–control studies that involved diverse populations at different infection stages (20, 21, 25–27). Compelling evidence points to the presence of SARS-CoV-2 viral protein in the brainstem, housing crucial cardiovascular control centers, even in cases of mild COVID-19 (28, 29). This phenomenon prompts consideration within the context of SARS-CoV-2 invading the vagal pathways, highlighting the intricate role of the nervous system in neuroimmunometabolism (30). Vagal sensory afferents innervating airways express transient receptor potential vanilloid 1 (TRPV1) and transient receptor potential ankyrin 1 (TRPA1), pivotal depolarizing calcium-permeable ion channels crucial in detecting environmental irritants and endogenous metabolites. Literature data consistently demonstrated that respiratory virus infection up-regulates TRPV1, TRPA1 receptors on airway cells (31) and lead to an increase in overall TRPV1 activation (32). This activation leads to neuropeptide release and neurogenic inflammation (33). In addition, our research group previously demonstrated that modulation of TRPV1 by inflammatory endogenous mediators changes cough sensitivity and autonomic regulation of cardiac rhythm in healthy subjects (34). All these evidences suggests that COVID-19 dysautonomia may stem from neuroinflammation and associated inflammatory conditions (35, 36).

Simultaneously, our findings reveal a noteworthy increase in sympathetic heart modulation, mirrored by elevated nLF and LF/HF, in individuals with mild SARS-CoV-2 infection consistent with observation in several case–control studies (22, 25, 26). However, this heightened sympathetic heart modulation may pose significant challenges for COVID-19 patients, as previously postulated by our group and others (9, 37, 38). The intertwining factors of aging and male gender, associated with sympathoactivation and linked with abdominal fat (39–41), might elucidate the increased risk of severe COVID-19 and related mortality in these demographics (42). Our investigation into sex differences in autonomic control of the heart unveiled a trend towards increased LF/HF in males compared to females, confirming literature data of a relative sympathetic dominance in male (43). Intriguingly, this sex difference attained statistical significance solely in the post-acute phase of infection, approximately one month from the negative SARS-CoV-2 NPS. This discrepancy did not persist in other follow-up timings, as detailed in Supplementary Table S4.

Our recent finding (34) pinpointed an *in vivo* mechanism operating in healthy subjects where sensitization of airway sensory TRPV1/A1 by endogenous mediators, such as prostaglandin-E_2_ (PGE_2_) and bradykinin (BK), disrupts autonomic cardiac rhythm, elevating sympathetic and suppressing vagal heart modulation. This cardiac autonomic imbalance, resembling that induced by SARS-CoV-2 in mild COVID-19 cases among HCWs, raises intriguing possibilities. While the direct interaction of SARS-CoV-2 with TRPV1/A1 receptors awaits investigation, increased levels of endogenous mediators during COVID-19, particularly elevated PGE_2_ in severe cases (44, 45) and dysregulated BK signaling (46) in patients with COVID-19 pneumonia (47), suggest potential involvement. Data from a single center cohort study showed that des-Arg9-bradykinin was significantly elevated in COVID-19 intensive care unit patients and was associated with disease severity (48). Furthermore, TRPV1/A1, implicated in the cough reflex, was confirmed in COVID-19 through induced cough challenges, demonstrating rapid relief with TRPA1/V1 agonists (green tea, curcumin, ginger, red pepper) (49, 50). Notably, various COVID-19 symptoms align with TRPV1/A1 channels (35, 36), reinforcing the likelihood of their role in the detected cardiac autonomic imbalance (35). In sensory neurons of mice (51), in rat dorsal root ganglion neurons (52) and in human corneal epithelial cells (53), activation of TRPV-1 unleashes pro-inflammatory substances like substance P (sP) and interleukin 6 (IL-6) respectively, key players in COVID-19 pathophysiology, with reported elevations correlating with illness severity (54, 55). Although inflammation levels were not measured, literature data hint at cardiac autonomic balance serving as a potential marker for identifying the neural pathways (parasympathetic and/or sympathetic) regulating inflammation (56), offering potential for early identification of subjects with long-COVID at risk of clinical deterioration (54, 57, 58). These data support the idea that desensitizing or blocking TRP channels could be a viable option for research into COVID-19 prevention and treatment (35, 36, 59, 60).

With respect to the clinical presentation, all group 1 HCWs reported at least three symptoms during the acute phase, and during follow-up visits, they consistently reported mild SARS-CoV-2 symptoms. The acute phase symptoms, including fever, myalgia/arthralgia, upper/lower airway symptoms, and headache, were comparable to those reported in a larger HCWs sample from the same hospital over an extended period (61). Persistent symptoms included fatigue, palpitations, dyspnea on exertion, and attention and memory problems. A systematic review of 194 studies conducted before January 2022 indicated that 45% of COVID-19 survivors, irrespective of hospitalization, experienced at least one unresolved symptom after an average follow-up of 126 days (62). Our data align with this, showing that 41.2% of HCWs continued to report symptoms at the 6-month follow-up. Recent data, including the Omicron wave, revealed a long-COVID prevalence between 24.0 and 30.3% among HCWs with previous SARS-CoV-2 infection (63, 64), in line with 37.8% of our HCWs reporting at least one symptom at the 13-month follow-up. Persistent symptoms of dysautonomia, such as fatigue, headache, palpitations, cough, dyspnea on exertion, and attention and memory problems, were prevalent in our population. Our findings resonate with a cohort study on 112 severe SARS-CoV-2 hospitalized patients, where 47% of long-COVID autonomic syndrome patients exhibited autonomic-related symptoms and reduced quality of life at 6 months and one-year follow-ups (65).

Our study has certain limitations. The sample size was relatively small limiting our inference potential. We intentionally excluded severe COVID-19 cases, which constitute only 20% of the total cases. The absence of inflammatory marker measurements during health surveillance visits will be addressed in a dedicated long-COVID study project. Most participants received booster vaccinations, making it impossible to draw conclusions about the role of vaccination based on our study design and results. Finally, we focused on post-COVID cardiac dysautonomia not considering the syndromic nature of autonomic dysfunctions, instead (10). However, our study’s strength lies in our innovative study design, where each subject serves as their own control, enhancing the validity of our findings. The selection of HCWs as a study population, minimize selection bias compared to patients referred to cardiology services, who may have higher symptom burdens. In addition, the predominance of mild cases with a better prognosis align with the general population trends, bolstering the relevance of our findings. Despite strict control over confounding factors such as night shifts, manual handling, comorbidities, and drug use, our results remain robust.

## 5 Conclusion

The most important findings can be summarized as follows.

SARS-CoV-2 associated autonomic imbalance (increased sympathetic heart modulation and decreased vagal heart modulation) in the post-acute phase after recovery of mild COVID-19, consistently resolved 6 months after the first negative SARS-CoV-2 NPS and in some HCWs already after two months. Significant reductions in mean HR occurred about one year after the negative SARS-CoV-2 NPS compared to the post-acute phase. However, mean HR always kept in the range of normal resting values. These results are consistent with epidemiological data suggesting a higher risk of acute cardiovascular complications in the first 30 days after COVID-19 infection. Therefore, in this early phase of infection HRV analysis could be helpful to identify patients at high risk of cardiac complications. However, time-series data collection is suggested, since there are currently no normative data for short-term measures of HRV. A significant proportion of HCWs reported long-term COVID-19 symptoms, which dot not seems to be related to cardiac autonomic balance, but provide an opportunity for a functional and clinical follow-up. Future research should certainly further evaluate the heterogeneity of COVID-19 patients to explore how subgroups of patients can have different trajectory of post-acute autonomic imbalance (66). Particular attention should be paid to TRPV1/A1 which might be involved in the pathogenesis of this cardiac autonomic imbalance. Further works are needed to test whether this autonomic imbalance have a role in the development of long-COVID syndrome.

## Data availability statement

The datasets presented in this article are not readily available because the data are not publicly available due to ethical and legal restrictions, as participants of this study did not agree for their data to be shared publicly. Requests to access the datasets should be directed to filippo.liviero@unipd.it.

## Ethics statement

The study was approved by the local Research Ethics Committee, Comitato Etico Territoriale Area Centro—Est Veneto (CET-ACEV)—Azienda Ospedale Università di Padova, (Protocol number = 267n/AO/22), and conducted in accordance with the ethical principles stated in the “Declaration of Helsinki”. Data were collected during routine health surveillance carried out in compliance with Legislative Decree 81/08 and European Community Directive 90/679. Written informed consent was obtained from all subjects involved in the study.

## Author contributions

FL: Writing – review & editing, Writing – original draft, Software, Methodology, Investigation, Formal analysis, Data curation, Conceptualization. MS: Writing – review & editing, Writing – original draft, Methodology, Data curation, Conceptualization. AV: Writing – original draft, Project administration. MB: Writing – original draft, Investigation, Formal analysis, Data curation. LF: Writing – original draft, Investigation, Formal analysis, Data curation. LB: Writing – original draft, Investigation, Formal analysis, Data curation. FF: Writing – review & editing, Writing – original draft, Visualization, Validation, Software, Resources, Methodology, Formal analysis, Data curation. AM: Writing – review & editing, Writing – original draft, Visualization, Validation, Supervision, Funding acquisition, Conceptualization. PM: Writing – review & editing, Writing – original draft, Visualization. SP: Writing – review & editing, Writing – original draft, Visualization, Supervision, Methodology.
