# Supplementary material for: Long term follow-up of heart rate variability in healthcare workers with mild COVID-19
Source: Front Neurol. 2024 May 17;15:1403551. doi: 10.3389/fneur.2024.1403551 (PMC11141692; doi:10.3389/fneur.2024.1403551)
Supplement: Supplementary file 1 [file Data_Sheet_1.docx]

**Supplementary Material**

**Table S1.** Absolute distribution and percentage of COVID-19 symptoms reported by subgroup A HCWs in the acute phase (baseline) and at 6-month follow-up. Statistical comparisons were made by Fisher exact test, level of significance < 0.05. Bold values indicate those that were considered as the most persistent symptoms (p > 0.05), at 6-month follow-up.

| Symptoms | Acute phase, n (%) | 6-month follow-up, n (%) | p-value |
| --- | --- | --- | --- |
| Fatigue | 15 (88.2) | 7 (41.2) | 0.01 |
| Myalgia | 14 (82.3) | 0 | < 0.0001 |
| Headache | 11 (64.7) | 1 (0.06) | 0.0008 |
| Arthralgia | 10 (58.9) | 1 (0.06) | 0.0024 |
| Cough | 10 (58.9) | 1 (0.06) | 0.0024 |
| Fever | 9 (52.9) | 0 | 0.0009 |
| Sore throat | 9 (52.9) | 0 | 0.0009 |
| Rhinorrhea | 7 (41.2) | 0 | 0.0072 |
| **Attention and memory problems** | **7 (41.2)** | **2 (11.8)** | **0.1175** |
| **Dyspnea on exertion** | **6 (35.3)** | **2 (11.8)** | **0.2245** |
| **Palpitations** | **6 (35.3)** | **3 (17.6)** | **0.4384** |
| Chest tightness | 5 (29.4) | 1 (0.06) | 0.1748 |
| Nausea and vomiting | 5 (29.4) | 1 (0.06) | 0.1748 |
| Ear pain | 4 (23.5) | 0 | 0.1026 |
| Nasal obstruction | 4 (23.5) | 0 | 0.1026 |
| Loss of taste | 4 (23.5) | 0 | 0.1026 |
| Mental fog | 4 (23.5) | 0 | 0.1026 |
| Anorexia | 4 (23.5) | 0 | 0.1026 |
| Insomnia | 2 (11.8) | 0 | 0.4848 |
| Burning eyes | 2 (11.8) | 1 (0.06) | 1.0000 |
| Loss of smell | 2 (11.8) | 0 | 0.4848 |
| Skin rash | 2 (11.8) | 0 | 0.4848 |
| Diarrhea | 2 (11.8) | 0 | 0.4848 |
| Tachycardia | 0 | 0 | 1.0000 |

**Table S2.** Absolute distribution and percentage of COVID-19 symptoms reported by subgroup B HCWs in the acute phase (baseline) and at 13-month follow-up. Statistical comparisons were made by Fisher exact test, level of significance < 0.05. Bold values indicate those that were considered as the most persistent symptoms (p > 0.05), at 13-month follow-up.

| Symptoms | Acute phase, n (%) | 13-month follow-up, n (%) | p-value |
| --- | --- | --- | --- |
| Myalgia | 25 (67.6) | 0 | < 0.0001 |
| Fatigue | 21 (56.8) | 4 (10.9) | < 0.0001 |
| Fever | 21 (56.8) | 0 | < 0.0001 |
| Arthralgia | 20 (54.0) | 1 (0.03) | < 0.0001 |
| Headache | 19 (51.4) | 2 (0.05) | < 0.0001 |
| Cough | 19 (51.4) | 1 (0.03) | < 0.0001 |
| Sore throat | 17 (45.9) | 1 (0.03) | < 0.0001 |
| Loss of taste | 16 (43.2) | 2 (0.05) | 0.0003 |
| **Palpitations** | **15 (40.5)** | **9 (24.3)** | **0.2140** |
| Rhinorrhea | 15 (40.5) | 0 | < 0.0001 |
| Loss of smell | 13 (35.1) | 2 (0.05) | 0.0029 |
| Chest tightness | 11 (29.7) | 2 (0.05) | 0.0123 |
| Dyspnea on exertion | 11 (29.7) | 0 | 0.0004 |
| Attention and memory problems | 9 (24.3) | 2 (0.05) | 0.0462 |
| Nasal obstruction | 7 (18.9) | 0 | 0.0114 |
| Nausea and vomiting | 7 (18.9) | 1 (0.03) | 0.0557 |
| Insomnia | 7 (18.9) | 0 | 0.0114 |
| Skin rash | 6 (16.2) | 0 | 0.0251 |
| **Mental fog** | **6 (16.2)** | **1 (0.03)** | **0.1070** |
| Burning eyes | 6 (16.2) | 0 | 0.0251 |
| Ear pain | 5 (13.5) | 0 | 0.0541 |
| Diarrhea | 5 (13.5) | 0 | 0.0541 |
| Anorexia | 3 (0.08) | 0 | 0.2397 |
| Tachycardia | 1 (0.03) | 0 | 1.0000 |

**Table S3.** Difference in the autonomic control of the heart, indexed by LF/HF among group 1 HCWs (subgroups A and B) at 6-mont and 13-mont follow-up, respectively. Two-sample t-test was used between HCWs with most significative persistent symptoms vs HCWs without significative persistent symptoms.

| Group 1 HCWs | LF/HF | Most significative persistent symptoms | No significative persistent symptoms | p-value |
| --- | --- | --- | --- | --- |
| Subgroup-A | Sex (m;f) | 1;4 | 2;10 |  |
|  | 6-month follow-up | 0.48 ± 0.40 | 0.78 ± 0.64 | 0.34 |
|  |  |  |  |  |
| Subgroup-B | Sex (m;f) | 2;8 | 8;19 |  |
|  | 13-month follow-up | 0.72 ± 0.70 | 1.15 ± 1.03 | 0.16 |

**Table S4.** Sex differences in the autonomic control of the heart, indexed by LF/HF among group 1 HCWs (subgroups-A, B and C) that were functionally followed at baseline (i.e. about 1 month) 6 months (subgroup-A), 13 months (subgroup-B), and 6 and 13 months (subgroup-C), after the negative SARS-CoV-2 NPS. Values are given as mean ± standard deviation. Two-sample t-test was used between males vs females HCWs. Bold values indicate statistically significant results (p< 0.05).

| Group 1 HCWs | LF/HF | Males, (n=3) | Females, (n=14) | p-value |
| --- | --- | --- | --- | --- |
| Subgroup-A | Baseline | 2.41 ± 0.54 | 1.09 ± 1.10 | **0.02** |
|  | 6-month follow-up | 1.52 ± 1.07 | 0.48 ± 0.30 | 0.24 |
|  |  | Males, (n=10) | Females, (n=27) |  |
| Subgroup-B | Baseline | 2.19 ± 1.70 | 1.33 ± 1.04 | 0.16 |
|  | 13-month follow-up | 1.70 ± 1.33 | 0.79 ± 0.66 | 0.06 |
|  |  | Males, (n=3) | Females, (n=10) |  |
| Subgroup-C | Baseline | 2.41 ± 0.54 | 1.29 ± 1.21 | 0.05 |
|  | 6-month follow-up | 2.38 ± 1.86 | 0.55 ± 0.34 | 0.23 |
|  | 13-month follow-up | 1.48 ± 0.43 | 0.86 ± 0.72 | 0.12 |

**Table S5.** Multiple regression analysis of the influence of elapsed days from the negative SARS-CoV-2 NPS, age, sex, night work, body mass index (BMI), cardiac symptoms (i.e. palpitations and tachycardia), systolic blood pressure difference (post-pre SARS-CoV-2 infection), diastolic blood pressure difference (post-pre SARS-CoV-2 infection) and manual handling of loads (MHL) and manual handling of patients (MHP) on delta LF/HF (post-pre SARS-CoV-2 infection). Bold values indicate statistically significant results (p< 0.05).

| **Variable** | **Coefficient** | **Standard error** | **t** | **p-value** |
| --- | --- | --- | --- | --- |
| **Elapsed days from the negative SARS-CoV-2 NPS** | -0.020 | 0.009 | -2.23 | **0.038 *** |
| **Age** | 0.007 | 0.014 | 0.52 | 0.610 |
| **Sex** | 0.236 | 0.343 | 0.69 | 0.499 |
| **Night work** | 0.503 | 0.301 | 1.67 | 0.111 |
| **BMI** | 0.037 | 0.044 | 0.85 | 0.408 |
| **Cardiac symptoms** | -0.164 | 0.357 | -0.66 | 0.651 |
| **Systolic blood pressure difference (post-pre)** | 0.004 | 0.015 | 0.27 | 0.787 |
| **Dyastolic blood pressure difference (post-pre)** | -0.021 | 0.025 | -0.84 | 0.411 |
| **MHL and MHP** | -0,077 | 0,246 | -0,31 | 0,757 |

**Table S6.** Sex differences in the autonomic control of the heart, indexed by LF/HF among group 2 HCWs at baseline (i.e. pre SARS-CoV-2 infection) and at about 1 month functional follow-up after the negative SARS-CoV-2 NPS. Two-sample t-test was used between males vs females HCWs. Bold values indicate statistically significant results (p< 0.05).

| Group 2 HCWs | LF/HF | Males, (n=7) | Females, (n=22) | p-value |
| --- | --- | --- | --- | --- |
|  | Baseline | 1.38 ± 0.46 | 0.91 ± 0.83 | 0.07 |
|  |  |  |  |  |
|  | 1-month follow-up | 2.06 ± 0.53 | 1.23 ± 1.34 | **0.03** |
